# Overlap Anastomosis for Digestive Reconstruction during Laparoscopic Distal Gastrectomy with Intensive Regional Lymph Node Dissection: Physiological Impact of Preserving the Mesenteric Autonomic Nerves in the Lifted Jejunal Limb

**DOI:** 10.1155/2018/4938341

**Published:** 2018-09-23

**Authors:** Taku Kitano, Daiki Yasukawa, Yuki Aisu, Tomohide Hori

**Affiliations:** Department of Digestive Surgery, Tenri Hospital, Tenri, Japan

## Abstract

Laparoscopic gastrectomy is a treatment for gastric cancer, and isoperistaltic side-to-side reconstruction is called “overlap anastomosis.” The physiological advantages of preserving the autonomic nerves in the jejunal limb for digestive reconstruction are well known. Here, we focused on overlap anastomosis with autonomic nerve-preserved mesojejunum of the lifted jejunal limb for laparoscopic distal gastrectomy with intentional lymph node dissection. Our surgical techniques and technical pitfalls were described in detail. The jejunum was partially sacrificed to preserve the autonomic nerves in the lifted jejunal limb. The length of the staple line was 35 – 40 mm. The endostapler entry was carefully closed to avoid even subtle stenosis. Twelve patients were retrospectively evaluated with a follow-up of 5.0 ± 0.6 years. Histological findings according to the Japanese classification were stage IA or IB. Dietary intake and postoperative ambulation occurred at 3.3 ± 1.0 and 1.3 ± 0.5 days after surgery, respectively. Postoperative complications according to Clavien–Dindo classification were one each of grade I and grade II. Postoperative hospital stay was 6.7 ± 1.6 days. Five patients were medication-free at final follow-up, with no recurrence in any patient. Overlap anastomosis with autonomic nerve-preserved jejunal limb was safe and feasible for laparoscopic distal gastrectomy with lymph node dissection.

## 1. Introduction

Laparoscopic gastrectomy for gastric cancer (i.e., total and distal gastrectomy accompanied with intensive regional lymph node dissection) is currently considered safe and feasible [1]. In Japan, based on definitive diagnoses according to the Japanese classification system [2], the Japanese guidelines [3] optimally indicate intentional dissection of regional lymph nodes as D1, D1+, D2, and D2+. Laparoscopic distal gastrectomy was first introduced in 1991 [4], and intracorporeal reconstruction has been adopted worldwide [5]. Although laparoscopic total gastrectomy has developed relatively slowly because of technical difficulties [6], intracorporeal side-to-side reconstruction based on an antiperistaltic approach (“functional end-to-end anastomosis” [7]) or an isoperistaltic approach (“overlap anastomosis” [8]) is now performed.

Reconstructive methods (i.e., Billroth I in 1881, Billroth II in 1885, and Roux-en-Y in 1893) were introduced in the late nineteenth century [9]. Although linear-stapled anastomosis for Billroth I (“delta anastomosis”) is suitable for laparoscopic distal gastrectomy [5], Billroth II or Roux-en-Y is required in patients with smaller gastric remnant or shorter duodenal stump. Although the disadvantages of vagotomy for Roux-en-Y reconstruction in total gastrectomy have been documented [10, 11], vagal nerves around the esophagus (Latarjet's nerves) are preserved during distal gastrectomy.

Intentional dissection of autonomic nerves around the celiac and superior mesenteric arteries is not required for early gastric cancer. The physiological advantages of preserving the autonomic nerves in the mesojejunum in the reconstructed alimentary limb have been documented [12, 13], and in our institution, we sacrifice the jejunum to create an autonomic nerve-preserved jejunal limb for Roux-en-Y reconstruction.

Functional end-to-end anastomosis [7] and overlap anastomosis [8] have been historically introduced for intracorporeal reconstructions during laparoscopic total gastrectomy. In our institution, these reconstructions were currently performed during laparoscopic total gastrectomy according to physician's choice. Contrastingly, Billroth I (delta anastomosis) [5], Billroth II, and Roux-en-Y are available for laparoscopic distal gastrectomy. As described above, Billroth II or Roux-en-Y is required in special situations. However, delta anastomosis has been accepted as the first choice in Japan [14, 15].

We retrospectively evaluated our experience with overlap anastomosis with autonomic nerve-preserved mesojejunum for the lifted jejunal limb in laparoscopic distal gastrectomy with intentional regional lymph node dissection. We also discuss the surgical techniques and technical pitfalls of this approach.

## 2. Patients and Methods

### 2.1. Critical Techniques for Reconstructive Surgery

**Figure 1:**
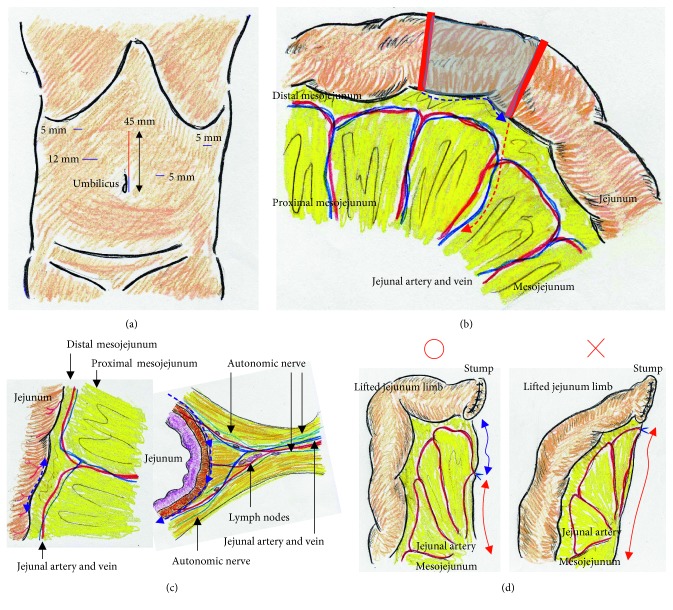
(a) Camera port positioned via the umbilicus; a total of five ports (blue lines) were placed. A 45 mm long incision extending from the umbilicus (red line) was made. (b) Approximately 20 cm of jejunum was sacrificed (shaded area), with the sacrificed length decided based on the segment's jejunal vessels (red solid lines). The jejunum was sacrificed (blue dotted line), and then, the mesojejunum was transected to lift the jejunal limb (red dotted line). (c) To preserve the distal mesojejunal autonomic nerves, the jejunum was sacrificed as close to the jejunal wall as possible (blue dotted line). Branches from the marginal vessels were carefully sealed in three groups using an advanced energy device to prevent both unexpected shortening of the mesojejunum and thermal nerve damage (blue dotted lines). (d) The sacrificed jejunum contributed well to ideal mesojejunal margins in the lifted jejunal limb (blue arrow). The jejunal limb could be lifted with no tension on the retracted mesojejunum (red arrows).

**Figure 2:**
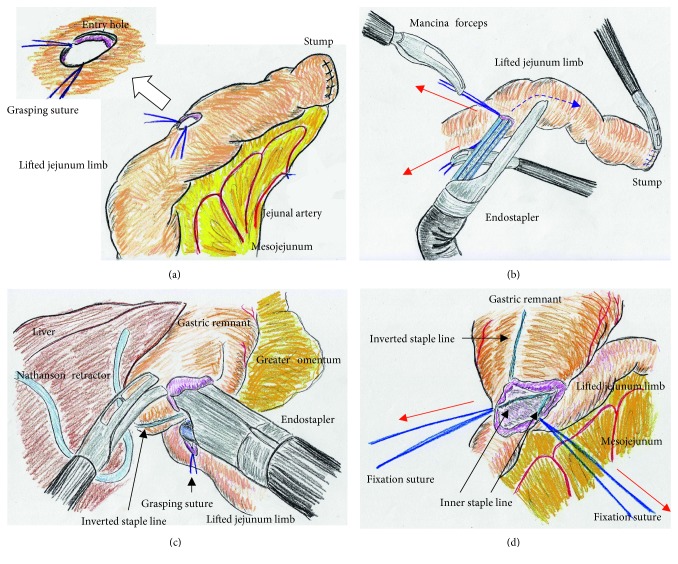
(a) The entry site for the endostapler was extracorporeally made. Two anchor sutures were preplaced. (b) Under countertraction by the two anchor sutures (red arrows), the endostapler was guided into the lifted jejunal limb (dotted blue line). (c) The endostapler was used side-to-side to attach the lifted jejunal limb to the gastric remnant in an isoperistaltic direction. (d) Two full-thickness anchor sutures (red arrows) were placed.

**Figure 3:**
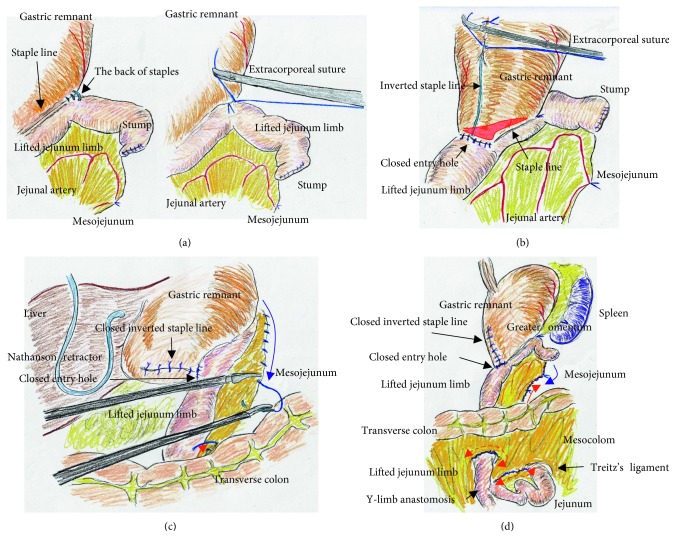
(a) A small number of seromuscular sutures were required at the tip of the endostapler. (b) We set the length of the staple line at 35–40 mm for ideal side-to-side stapled anastomosis (red area). The endostapler entry site was carefully closed, and the inverted staple line in the gastric remnant was covered. (c) Through a small laparotomy, gaps in the mesojejunum could be closed even along the upper side of the mesocolon (red arrow). Therefore, intracorporeal sutures were required to close gaps in the mesojejunum only along the upper side of the mesocolon (blue arrow). (d) Through a small laparotomy, gaps in the mesojejunum from Treitz's ligament (dotted red arrow) and the lower side of the mesocolon along the retrocolic route (dotted red arrow) could be closed. Intracorporeal sutures to close gaps in the mesojejunum were minimized (blue solid arrow) because of the closure along the upper side of the mesocolon through the small laparotomy (red solid arrow).

**Figure 4:**
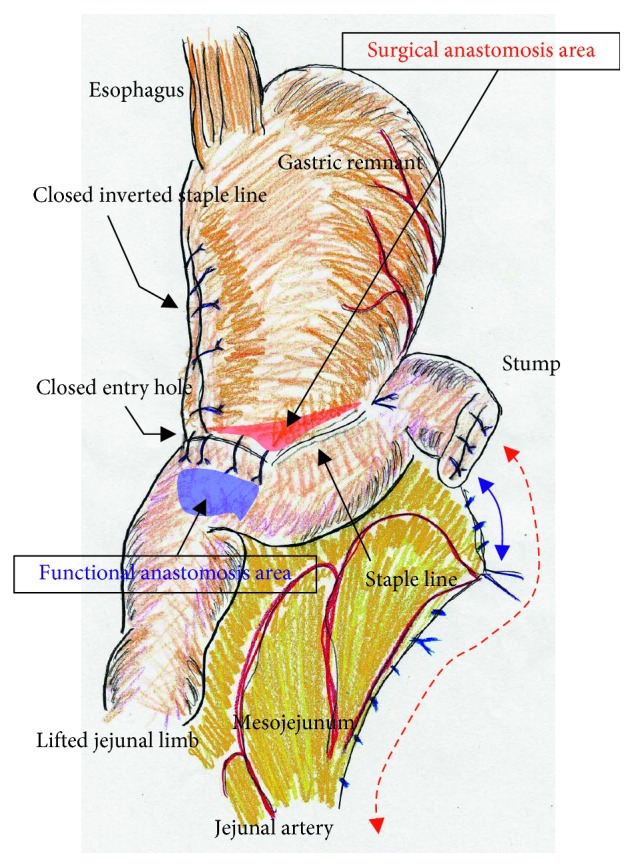
Overlap anastomosis involves linear-stapled reconstruction using an isoperistaltic side-to-side approach. Functional and surgical anastomoses are distinct for overlap anastomosis, and postoperative passage depends on patency at the functional anastomosis (blue area), not on the length of the staple line at the surgical anastomosis (red area). The endostapler entry site was carefully closed with layer-to-layer sutures using running sutures in the mucosa and interrupted sutures in the seromuscular layer using absorbable monofilament suture. Longer staple lines may result in a postoperative pouch-like dilatation in the lifted jejunal limb; therefore, we set the length of the staple line at 35–40 mm. A defect behind the staples at the tip of endostapler was closed. Inverted staple lines were covered. Even subtle tension on the mesojejunum was avoided as much as possible by sacrificing the jejunum (blue solid arrow), and a well-defined mesojejunum in the lifted jejunal limb (red dotted arrow) effectively preserved the mesenteric autonomic nerves. Mesenteric gaps were closed routinely.

**Figure 5:**
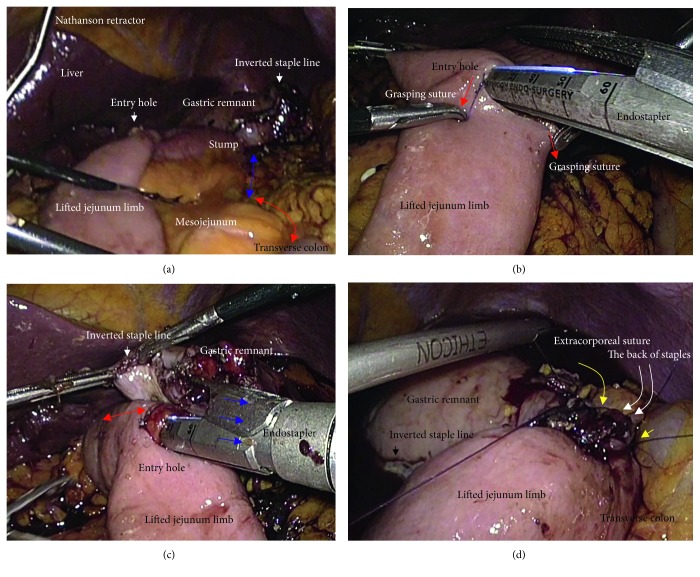
Actual findings via laparoscope during intracorporeal procedures were shown. Co-author, (T) Hori, mainly performed this surgery. (a) Two full-thickness anchor sutures (red arrows) were bilaterally placed for secure closure of the entry site. (b) Mucosal layers at the entry site were closed (red arrow). (c) Seromuscular layers at the entry site were closed. (d) Seromuscular sutures were made to cover the back of staples (yellow arrows).

**Figure 6:**
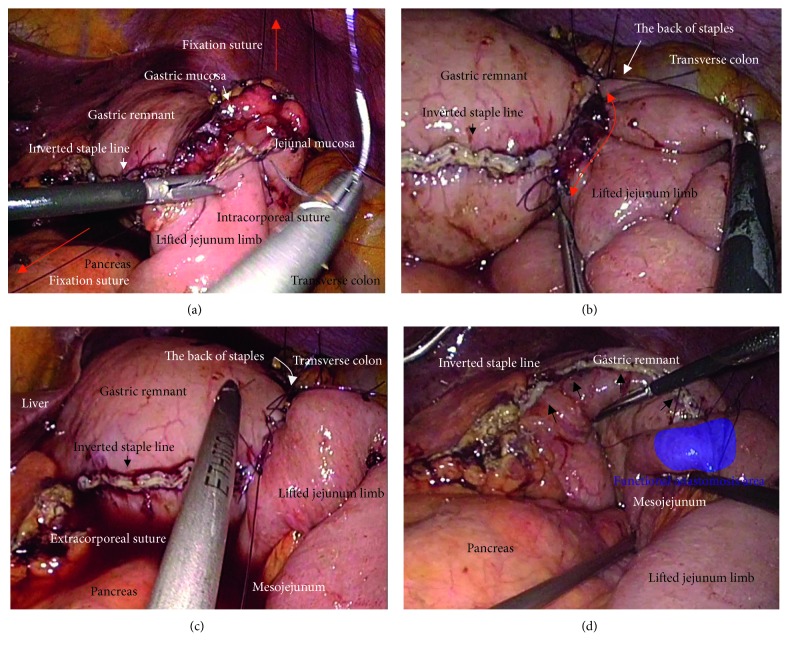
Actual findings via laparoscope during intracorporeal procedures were shown. Co-author, (T) Hori, mainly performed this surgery. (a) The jejunum was lifted through the antecolic route. The sacrificed jejunum contributed well to ideal mesojejunal margins in the lifted jejunal limb (blue arrow). The jejunal limb could be lifted with no tension on the retracted mesojejunum (red arrow). (b) Under countertraction by the two grasping sutures (red arrows), the endostapler was guided into the lifted jejunal limb. (c) Staple lines for surgical anastomosis should be set at 35–40 mm (red arrow). The length of linear staple line was optimally adjusted in the isoperistaltic side-to-side fashion (blue arrows). (d) Postoperative passage depends on patency at the functional anastomosis (blue area). The inverted staple line in the gastric remnant was covered (yellow arrows).

We placed a total of five ports. The lateral segment of the liver was retracted using a Nathanson's retractor. We extended the incision from the umbilicus for 45 mm (Figure 1(a)) and extracted the resected stomach through a small laparotomy incision. A suitable point from Treitz's ligament for Roux-en-Y anastomosis was marked beforehand, and the marked jejunum was retracted outside the body. Approximately 20 cm of the jejunum was sacrificed, with the sacrificed area determined based on the segment's jejunal arteries and veins (Figure 1(b)). To preserve autonomic nerves in the distal mesojejunum, the jejunum was sacrificed as close to the jejunal wall as possible. Branches from the marginal vessels were carefully sealed at the jejunal wall. The mesojejunum was usually dissected in three sections using an advanced energy device (Harmonic Ace +7; Ethicon, Cincinnati, OH, USA) to prevent both unexpected shortening of the mesojejunum and thermal nerve damage (Figure 1(c)). Using this approach, the jejunal limb could be lifted without tension on the elevated mesojejunum (Figure 1(d)), which provided better conditions for the mesenteric autonomic nerves. The stump of the lifted jejunal limb was covered by interrupted seromuscular sutures. The entry site for the endostapler was made extracorporeally at the estimated point for both an adequate length of isoperistaltic stapling and a suitable length from the end of the staple line to the jejunal stump. For subsequent procedures during intracorporeal anastomosis, two anchor sutures were preplaced at the relatively distal side of the endostapler entry site (i.e., the side opposite the direction of endostapler insertion) (Figure 2(a)). Next, part of the extended incision was closed before resuming pneumoperitoneum because an excellent surgical field was required for intracorporeal digestive reconstruction (Figure 1(a)). Under countertraction with the two anchor sutures, the endostapler (GST system (blue cartridge, 45 mm) and Powered Echelon Flex, Ethicon) was guided into the lifted jejunal limb through the entry site (Figure 2(b)). The endostapler was set for side-to-side use to staple the lifted jejunal limb to the gastric remnant in an isoperistaltic direction and then clamped. Clamped tissues were compressed for 2 minutes before stapling (Figure 2(c)) because this endostapler has an advantage for secure stapling with tissue precompression [16, 17]. The endostapler was fired, and hemostasis was completed using a soft-coagulation device if needed, taking care to ensure that the mucosa or stapling edge was inverted. Next, two full-thickness anchor sutures were placed for secure closure of the entry site and to bilaterally clarify termination points for subsequent sutures (Figure 2(d)). The entry site was closed with layer-to-layer sutures (i.e., running sutures in the mucosa and interrupted sutures in the seromuscular layer) using absorbable monofilament suture (Monocryl, 3-0, violet, 90 cm; Ethicon). At the tip of the endostapler, we easily saw an opening behind the staples, and a couple of seromuscular sutures were required to prevent postoperative leakage (Figure 3(a)). Longer staple lines may result in a postoperative pouch-like dilatation of the lifted jejunal limb and subsequent ileus in this dilatation. Therefore, the length of the staple line was set at 35–40 mm (Figure 3(a)). The entry site should be carefully closed to avoid even subtle stenosis; therefore, we used layer-to-layer sutures to close the entry site. Inverted staple lines in the gastric remnant were covered by interrupted seromuscular sutures (Figure 3(b)). Through a small laparotomy before closure, we were able to create a Y-limb anastomosis approximately 30 cm distal to Treitz's ligament and close gaps in the mesojejunum from Treitz's ligament and the lower side of the mesocolon along the retrocolic route. We could also close gaps in the mesojejunum of the lifted jejunal limb even along the upper side of the mesocolon. Intracorporeal sutures to close gaps in the mesojejunum of the lifted jejunal limb were required only along the upper side of the mesocolon (Figure 3(c) and 3(d)). It is critical to recognize that functional and surgical anastomoses are distinct for overlap anastomosis. Postoperative passage depends on patency at the functional anastomosis, not on the length of the staple line at the surgical anastomosis. Even subtle tension on the mesojejunum was avoided by sacrificing part of the jejunum, and a well-defined mesojejunum preserved the autonomic nerves in the mesojejunum of the lifted jejunal limb. Mesenteric gaps were closed with nonabsorbable sutures (Prolene, 3-0, SH-1; Ethicon) (Figure 4). Knot tying can be performed either intra- or extracorporeally based on surgeons' preference, and if possible, a leak test should be performed following anastomosis. Finally, the tip of a drainage tube was placed behind the anastomosis site. Actual findings during laparoscopic surgery are shown in Figures 5 and 6.

### 2.2. Patients

From April 2012 to March 2014, we performed overlap anastomosis for digestive reconstruction during laparoscopic distal gastrectomy with intensive regional lymph node dissection and are following current postoperative conditions in 12 patients. Mesenteric autonomic nerves in the lifted jejunal limb were preserved. Patients' profiles are summarized in Table 1. The following data are shown as mean and standard deviation: The average age at surgery was 65.4 ± 13.9 years, and patients included seven men and five women. All patients underwent preoperative enhanced computed tomography for metastasis survey and endoscopic ultrasonography for depth assessment, and preoperative diagnoses of stage I were made according to the Japanese classification system [18]. Operative time was 294.1 ± 35.1 minutes, and blood loss was 60.8 ± 48.1 ml. Definitive diagnoses based on histological findings of resected specimens according to the Japanese classification system [18] included five cases of T1bN0M0 pStage IB, four of T1aN0M0 pStage IA, two of T2N0M0 pStage IB, and 1 of T1bN1M0 pStage IB. According to the Japanese guidelines [19], intentional regional lymph node dissection was performed in seven D2 dissections and five D1+ dissections. Drains were removed on postoperative day 2.9 ± 1.0.

## 3. Results

### 3.1. Short-Term Courses and Long-Term Outcomes

To shorten postoperative times to adequate meal intake and sufficient ambulation [20], both rehabilitation counselors and physical therapists intensively intervened from postoperative day 1. Sufficient dietary intake and postoperative ambulation occurred on postoperative days 3.3 ± 1.0 and 1.3 ± 0.5, respectively. One patient experienced surgical site infection and one experienced intraperitoneal fluid collection, and postoperative complications according to the Clavien–Dindo classification [21, 22] were categorized as 1 each of grade I and grade II. Because deep venous thrombosis can readily develop perioperatively with laparoscopic surgery, prophylaxis for deep venous thrombosis was routinely performed with low-molecular-weight heparin from postoperative day 1 to hospital discharge, according to patients' risk assessments [23]. Postoperative hospital stay was 6.7 ± 1.6 days.

The follow-up term was 5.0 ± 0.6 years. Body weight loss compared with preoperative weight was 7.4 ± 1.6 kg. Although seven patients received medications (e.g., digestive enzyme, antiflatulent, and aperient), five patients required no medications, and no recurrence was seen in any patient.

## 4. Discussion

Laparoscopic partial gastrectomy has a similar oncological outcome to open resection and some advantages regarding lower intraoperative stress, earlier meal ingestion, less postoperative pain, better cosmesis, earlier ambulation, earlier hospital discharge, and better quality of life [6, 24, 25]. The techniques for distal gastrectomy, laparoscopic surgery, technical procedures for intensive regional lymph node dissection, and intracorporeal linear-stapled anastomosis for Billroth I gastrectomy are well established [4, 5, 26]. However, in patients unsuitable for intracorporeal linear-stapled anastomosis for Billroth I gastrectomy, the Roux-en-Y method is required because Roux-en-Y provides better postoperative quality of life with lower incidences of bile reflux and anastomotic leakage [27]. Postoperative function following Roux-en-Y reconstruction in distal gastrectomy has been well investigated for the retrocolic and antecolic routes [28, 29]. Although studies show that the retrocolic route may be superior to antecolic reconstruction [28, 29], we chose the retrocolic route to minimize tension on the mesenteric autonomic nerves in the lifted jejunal limb.

Overlap anastomosis was first described for linear-stapled reconstruction during laparoscopic total gastrectomy using an isoperistaltic side-to-side approach [8]. A lifted jejunal limb is required for overlap anastomosis, and a well-designed surgery is important for successful gastrojejunostomy. In our experience with conventional open distal gastrectomy with overlap anastomosis, longer staple lines of 60 mm resulted in a postoperative pouch-like dilatation near the surgical anastomosis in the lifted jejunal limb, and stagnation in this dilatation disturbed passage through the functional anastomosis; postoperative symptoms were intractable in these patients. We suggest that staple lines for surgical anastomosis should be set at 35–40 mm. An important concept is that functional and surgical anastomoses are distinct in creating the ideal design for overlap anastomosis.

Roux-en-Y stasis syndrome has been reported [29, 30], as well as the disadvantages of vagotomy in Roux-en-Y reconstruction [10, 11] because vagal nerves around the esophagus are usually transected in total gastrectomy. However, vagal nerves around the esophagus are preserved during distal gastrectomy; therefore, we focused on the benefits of a sacrificed jejunum when creating the lifted jejunal limb [12, 13]. Intentional dissection of autonomic nerves around the celiac and superior mesenteric arteries is not required for early gastric cancer, although these nerves may be sacrificed because of direct invasion in locally advanced cases. Overall, a lifted jejunal limb with autonomic nerve-preserved mesojejunum may provide an excellent postoperative course after distal gastrectomy, especially in early gastric cancer without intentional dissection of autonomic nerves.

Petersen first reported postoperative internal hernia after reconstruction with antecolic Billroth II gastrectomy in 1900 [31]. Internal hernia of both the mesenteric and Petersen's defects has the lowest incidence following laparoscopic Roux-en-Y gastric bypass [32, 33], although we experienced some cases of internal hernia into either a mesenteric or Petersen's defect after laparoscopic gastrectomy when we were not closing these defects [34]. Mesenteric and Petersen's defects should be closed using nonabsorbable sutures [35]; after we made this change, we experienced no postoperative internal hernia.

Suturing the mucosa separately (i.e., double-layered or layer-to-layer anastomosis) may be not the common practice for the gastrojejunal anastomosis. It is reported that the rate of postoperative leakage is lower in the double-layered anastomosis than the simple single-layered anastomosis [36, 37]. Moreover, during a gastrojejunostomy, a hand-sewn double-layered anastomosis has an advantage for a prevention of postoperative stenosis [38]. Hence, we employed a hand-sewn double-layered anastomosis, because a potential benefit for gastrojejunostomy had been documented [36–38].

A limitation of our study is the retrospective, single-institution design, which may be affected by a number of biases and primarily, selection bias. Therefore, we understand that conclusions must be drawn with extreme caution. We used overlap anastomosis with autonomic nerve-preserved mesojejunum with a lifted jejunal limb during laparoscopic distal gastrectomy, and we suggest that this isoperistaltic reconstruction with nerve preservation may have beneficial physiological potential.

## 5. Conclusion

Overlap anastomosis with an autonomic nerve-preserved jejunal limb is safe and feasible for laparoscopic distal gastrectomy with intentional lymph node dissection.

## Figures and Tables

**Table 1:** Patient Profiles.

Operative time (minute)	Blood loss (ml)	Lymph node dissection^*∗*^ (*D*)	Histopathological diagnosis^*∗∗*^			Drain removal (POD)	Dietary intake (POD)	Ambulation (POD)	Hospital discharge (POD)	Body weight loss (kg)	Complications ^*∗∗∗*^(grade)	Medication (Yes/No)
			(*T* factor)	(*N* factor)	(Stage)							
341	20	1+	1a	0	IA	1	2	1	4	2	—	Yes
292	40	2	1b	0	IA	2	3	1	6	7	—	Yes
270	60	1+	1a	0	IA	4	4	1	7	10	—	No
266	0	2	2	0	IB	3	4	2	8	17	—	No
302	60	2	1b	0	IA	3	4	1	7	1	—	Yes
287	110	1+	1a	0	IA	2	4	2	8	2	—	Yes
312	100	2	1b	1	IB	3	3	1	6	0	—	No
286	150	2	2	0	IB	4	4	1	8	7	I	Yes
272	0	1+	1a	0	IA	3	2	2	7	12	—	No
243	110	2	1b	0	IA	2	5	2	9	18	II	Yes
289	20	2	1b	0	IA	4	3	1	6	8	—	Yes
374	60	1+	1b	0	IB	4	2	1	4	5	—	No

^*∗*^Japanese gastric cancer treatment guidelines. ^*∗∗*^Japanese classification of gastric carcinoma. ^*∗∗∗*^The Clavien-Dindo classification. Abbreviation: POD; postoperative day.

## Data Availability

In our paper, all detailed data of each patient are clarified in Table 1. Second analyses are possible according to data in the table.
